# Adult Dermatomyositis with Bleeding Ulcer in the Pharynx

**DOI:** 10.1155/2014/854841

**Published:** 2014-09-22

**Authors:** Junko Kusano, Yuka Takahashi, Yoshikata Misaki, Norihiko Murai

**Affiliations:** ^1^Department of Otolaryngology, Kyoto-Katsura Hospital, Yamada Hirao-cho 17, Nishikyo-ku, Kyoto 615 8256, Japan; ^2^Department of Rheumatology, Kyoto-Katsura Hospital, Yamada Hirao-cho 17, Nishikyo-ku, Kyoto 615 8256, Japan

## Abstract

Dermatomyositis (DM) is one of the idiopathic inflammatory myopathies caused by complement-mediated vasculopathy or vasculitis in the muscle. Although the gastrointestinal (GI) mucosa has been reported to be involved as a result of vasculitis or vasculopathy, ulceration in the pharynx is a rare manifestation of DM. A 54-year-old woman complaining of muscle weakness in the extremities, low-grade fever, and dysphagia was diagnosed as having DM. Despite medical treatment with corticosteroids and immunosuppressive agents, her DM progressed rapidly, leading to exacerbation of the dysphagia. About 3 weeks after undergoing tracheostomy as a preventive measure against aspiration, the patient developed intractable respiratory tract hemorrhage. Repeated laryngoendoscopy revealed a bleeding ulceration in the pharynx that required hemostasis with electric cautery under general anesthesia. No bleeding recurred thereafter. Histopathologically, the pharynx exhibited nonspecific inflammatory cell infiltration in the muscle tissue. This rare manifestation may be considered in cases of DM with unexplainable airway bleeding.

## 1. Introduction

Dermatomyositis (DM) is one of the idiopathic inflammatory musculopathies with both systemic and autoimmune nature, characterized by bilateral symmetrical proximal muscle weakness and pain, specific skin lesion, and positive autoantibodies [1, 2]. Well-known manifestations in the otolaryngological area include dysphagia and dysarthria attributed to pharyngeal muscle involvement [3]. However, it is very rare that the pharyngeal mucosa exhibits ulcer, bleeding, or perforation [4]. Herein, we report a case of adult DM complicated with a bleeding pharyngeal ulcer.

## 2. Case Presentation

**Figure 1:**
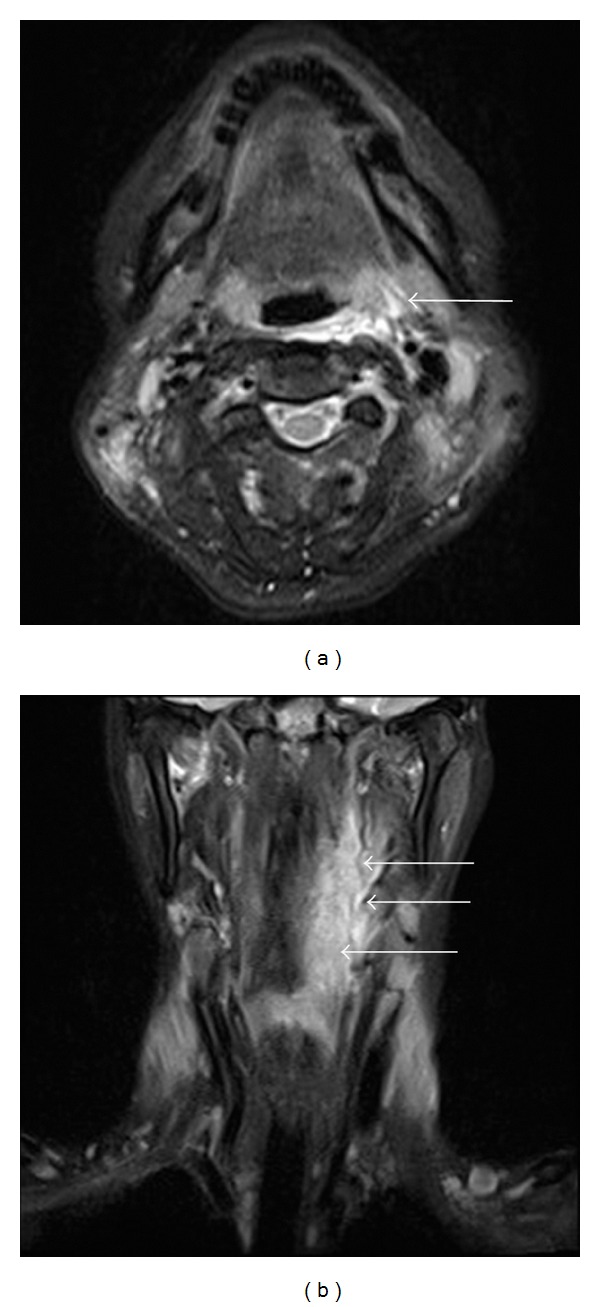
MRI of the neck: (a) T2WI axial section and (b) T2WI coronal section T2 hyperintensities are observed in the bilateral neck muscles, predominantly in the left pharyngeal constrictor muscles (arrows).

**Figure 2:**
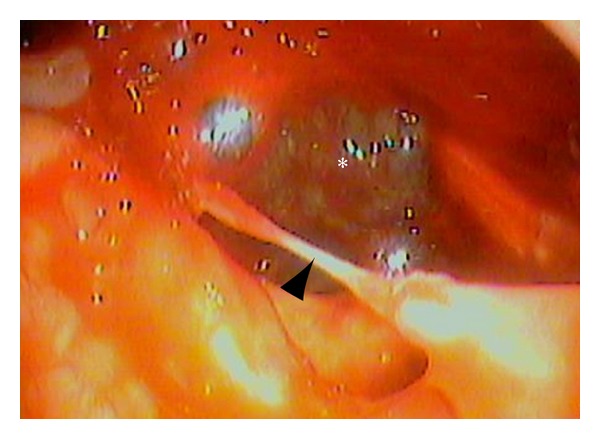
A fiberscopic image showing the bleeding ulcer in the left lateral to the posterior part of the oropharynx. Note that the left of the figure indicates the medial side, lower anterior, since this is a view through an otolaryngological pharyngolaryngeal fiberscope. An ulcer is found from the left lateral wall of the oropharynx to the hypopharynx (asterisk). A clot is observed within the ulcer. The remnant mucosa in the ulcerated area formed a bridge-like structure (arrow heads).

**Figure 3:**
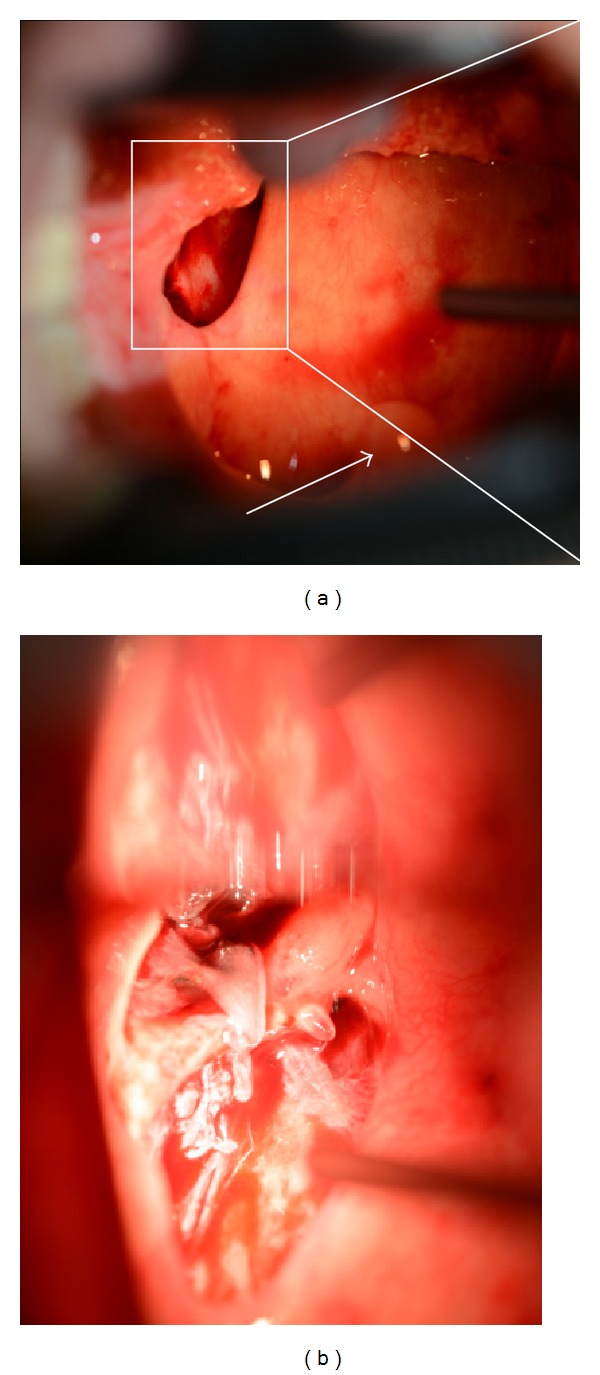
Intraoperative macroscopic findings of the left lateral and posterior walls of the oropharynx. Note that the left sides of the figures indicate the lateral side, lower cranial, since these photographs are taken through oral specula for tonsillectomy. (a) Inset shows the ulcerated area. Arrow indicates the uvula. (b) An enlarged view of the ulcer. The coagulated material and necrotic tissue are removed. No pus or tumorous lesions are noted.

**Figure 4:**
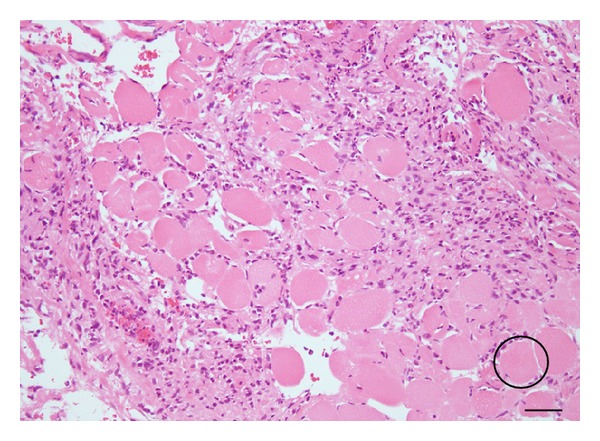
Histopathological findings of the pharyngeal constrictor muscle obtained during surgery. Bar: 50 *μ*m. Inflammatory cell infiltration is observed among the striated muscle fibers (circle).

A 55-year-old woman complaining of bilateral symmetrical proximal muscle weakness in the extremities, low-grade fever, and dysphagia that progressed for 10 days was referred by her family physician and admitted to the Department of Rheumatology, Kyoto-Katsura Hospital. On physical examination, facial erythema was observed spreading from the root of the nose to the bilateral cheeks, while Gottron's sign or heliotrope rash was absent. Blood examination revealed elevation of CK, AST, LDH, and CRP but no leukocytosis. Antinuclear antibody was positive (×40) whereas all antibodies against Jo-1, DNA, SS-A/Ro, SS-B/La, PR3-ANCA, and MPO-ANCA were negative. T2-weighted MRI showed hyperintensities in the bilateral neck muscles including the left pharyngeal constrictor muscle (Figure 1), as well as the bilateral iliopsoas and femoral muscles. Biopsy from the left vastus lateralis muscle demonstrated lymphocyte infiltration in the endomysium and vacuolar degeneration of the muscle fibers, but specific findings of DM, such as perivascular mononuclear infiltration and perifascicular atrophy and inflammation, were absent. On the other hand, the facial erythema exhibited perivascular lymphocytic infiltration and mucin deposits. On the basis of the clinical symptoms, laboratory data, skin histopathology, and radiologic findings of the muscles, the diagnosis of DM was established. Four days after admission, otolaryngology was consulted regarding the dysphagia. On pharyngolaryngeal fiberscopy, elevation of the larynx was moderately impeded, and slight retention of the saliva in the pyriform sinuses was noted, but gross movement of the vocal cords and pharyngeal sensation remained intact with no organic mucosal lesions. Medical therapy with corticosteroid was commenced on the 7th day of hospitalization, with an initial dose of 55 mg/day (1 mg/kg) prednisolone, which was increased to 80 mg/day due to a poor response by the 4th week after admission. Regarding deglutition, she was able to orally take over half of a softened meal, but the dysphagia progressed rapidly despite intensive medical treatment. On the 4th week, intravenous hyperalimentation was started since she was no longer able to swallow meals or fluids. Gait disorders deteriorated as well, resulting in the patient becoming completely bedridden by the 25th day. In consideration of the rapidly progressive nature of her disease, she elected to undergo tracheostomy as a preventive measure against aspiration pneumonia on the 31st day. Thereafter, by the 8th week after admission, the corticosteroid was switched to betamethasone (12 mg/day), and immunosuppressants (tacrolimus: 2.0 mg/day p.o., 21st–30th day; cyclophosphamide: 700 mg/day, 32nd and 57th day; cyclosporine: 150 mg/day, 34th–36th day) and immunoglobulin were added subsequently (22.5 g/day, 21st–25th day and 49th–53rd day) due to the persistent lack of response and further disease progression. During this refractory disease course, on the 56th day after admission (the 25th day after tracheostomy), the patient developed massive hemoptysis from the tracheostomy tube and the mouth. Chest CT revealed a ground glass appearance in the right lower lobe of the lung and soft tissue densities in the peripheral airways suggesting aspiration of the bloody secretion. Otolaryngological examination including endoscopic observation of the tracheostoma, lower airway, and subglottic space failed to reveal any source of the bleeding. Transnasal fiberscopic inspection of the upper airway on the 59th day of hospitalization revealed retention of a mixture of coagulated and fresh blood in the left lateral wall of the oropharynx, with the caudal end reaching the hypopharynx (Figure 2). Suction removal of the debris demonstrated intermittent bleeding from this site. On the following day, hemostatic procedures were undertaken transorally under general anesthesia. After removal of the coagulated debris and collection of biopsy specimens from the lesion via oral specula for tonsillectomy (Figure 3), the bleeding was treated with bipolar and monopolar electric cautery, followed by closure with absorbable sutures and ligations. Absorbable hemostatic particles (Arista AH, Medafor, Inc., USA) and a microfibrillar collagen hemostat (Avitene, Davol Inc., USA) were also applied. The hemoptysis stopped immediately after the hemostatic procedures. Bacterial examination was negative for pathological organisms, including acid-fast bacteria. Histopathologically, inflammatory cellular infiltration was evident among the muscle fibers, but there were no signs of perivascular lymphocytes or perifascicular atrophy and inflammation (Figure 4). The pharyngeal wound healed well resulting in complete epithelialization, and the patient was able to proceed to deglutition rehabilitation 3 months after the surgical hemostasis. No recurrence of the bleeding was noted up to the 6th month after surgery.

## 3. Discussion

The mechanism underlying the development of DM involves the activation of complement cascades by autoantibodies targeting the endothelium of endomysial capillaries and microvessels, leading to vasculopathies of muscular microvessels, necrosis of the capillary vessels, perivasculitis, and ischemic myofiber damage, which is mediated by a complement membrane attack complex. Histopathologically, myofibers develop mononuclear infiltration and necrosis followed by regeneration [1–3]. The age distribution of DM onset is bimodal with one peak between 5 and 15 years and the other from 50 to 60. Juvenile-onset DM is characterized by more extensive necrotic vasculitis, resulting in a greater variety of skin lesions and other clinical symptoms, such as GI ulceration and/or perforation and neurological symptoms, compared with adult-onset DM. In consideration of this difference, when the classification system of DM was proposed by Bohan and Peter in 1975, the juvenile-onset type was classified as “childhood dermatomyositis” [5]. While the incidence of ulceration and bleeding perforation of GI tracts in childhood DM is as low as 2-3%, the incidence in adult DM is even lower (~1%) [6]. The reported sites for GI involvement in adult DM include the esophagus [7–9], duodenum [10, 11], and intestine [8]. Another example of perforation of the hollow organs is mediastinal emphysema that has originated from a tracheal perforation, which is considered to be related to vasculitis [12, 13].

In most cases, pharyngeal manifestations of DM include dysarthria and dysphagia due to pharyngeal muscle weakness. Ulceration and perforation of the pharynx is a very rare manifestation of DM, with only one case from France having been reported to date [4]. That case exhibited dysphagia about 10 months after onset, and the pharyngeal lesion proceeded to ulceration with exposure of the prevertebral fascia. The histopathological findings included ulcerations of the mucosa with intravascular thrombosis and necrosis and fascicular inflammatory infiltration. Laboratory data showed elevated CK, AST, and LDH but no leukocytosis, with only slight positivity for antinuclear antibody. All antibodies against Jo-1, DNA, SS-A, SS-B, and Scl-70 were negative. Muscle biopsy revealed muscle fiber necrosis, disappearance of myofibrils, and thrombosis predominantly in the capillary vessels in the endomysium. The case was treated with prednisolone and cyclophosphamide. The French case and ours share some common features, which include the presence of dysphagia, resistance to medical therapies, and a single positive antinuclear antibody. However, the present case is distinct in that ours showed a more rapid progression and lacked any histopathological evidence of vasculopathy in the ulcerative lesion. This does not necessarily exclude the possibility of the pharyngeal lesion being a manifestation of DM. In the reported cases of adult-onset DM with gastrointestinal (GI) involvement, it is not uncommon for the ulcer specimens to display only nonspecific inflammatory findings. It is possible that medical therapies with steroids and immunosuppressive agents could obscure the vasculitis in the perforated lesions. In fact, histopathological evidence of vasculopathy was identified in the GI lesions in only three out of six of the aforementioned cases of adult-onset DM with GI involvement. In the remaining cases, the GI lesions were regarded as a manifestation of DM based on the fact that there were no signs of any infectious source, the GI sites involved were atypical for steroid-induced ulceration, and the vasculopathies were demonstrated in the skin lesion.
